# Fournier’s Gangrene Treated with Perianal Loop Drainage and Aggressive Surgical Debridement while Preserving Postoperative Anal Sphincter Function: A Case Report

**DOI:** 10.70352/scrj.cr.25-0482

**Published:** 2025-12-03

**Authors:** Ryosuke Aoki, Wataru Adachi, Jiro Imura, Yoshiaki Haba

**Affiliations:** Department of Surgery, Fujimi-Kogen Hospital, Fujimi-Kogen Medical Center, Suwa, Nagano, Japan

**Keywords:** Fournier’s gangrene, anal sphincter dysfunction, loop drainage technique

## Abstract

**INTRODUCTION:**

Fournier’s gangrene is a rapidly progressive necrotizing fasciitis affecting the external genitalia and perineal region, with a poor prognosis and mortality rate of up to 20%. Prompt infection control, typically through extensive debridement, is essential for survival but may impair anal sphincter function and patient’s QOL. In the present case, infection control and anal sphincter preservation were successfully achieved using the loop drainage as the initial treatment strategy.

**CASE PRESENTATION:**

A 77-year-old man with a history of alcoholic liver cirrhosis and cerebral infarction presented with fever, redness, and swelling around the anus. Fournier’s gangrene was diagnosed based on physical examination and imaging findings, and emergency debridement was performed. A loop drainage technique using Penrose drains was used around the anus to preserve anal function. Postoperatively, additional debridement and drainage procedures resulted in successful infection control. No postoperative defecatory dysfunction was observed, and the patient was discharged with a preserved QOL.

**CONCLUSIONS:**

The loop drainage technique using Penrose drains may be an effective therapeutic option for perianal involvement in Fournier’s gangrene.

## Abbreviations

FG
: Fournier’s gangrene
FGSI
: Fournier’s Gangrene Severity Index
UFGSI
: Uludag Fournier’s Gangrene Severity Index

## INTRODUCTION

FG is a rapidly progressive necrotizing fasciitis affecting the external genitalia and perineal region, with a poor prognosis and mortality rate of up to 20%.^1,2)^ Prompt infection control, often requiring extensive surgical debridement, is critical for survival. However, extensive surgical debridement may lead to significant functional deficits.^3)^

This study reports a case of successful infection control and preservation of postoperative anal function in FG using a loop drainage technique as the initial treatment strategy.

## CASE PRESENTATION

A 77-year-old man with a history of alcoholic liver cirrhosis and cerebral infarction, under regular follow-up at our hospital’s Department of Gastroenterology, presented with fever for 1 day, along with redness and swelling around the anus. On presentation, his vitals were body temperature 38.9°C, blood pressure 104/64 mmHg, and pulse rate 104 beats per minute.

**Fig. 1:**
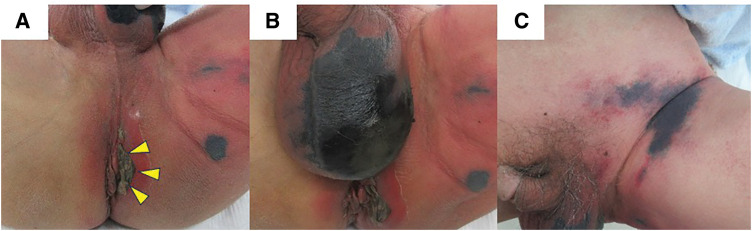
Marked erythema with necrotic changes observed at the 3 and 6 o’clock positions in the perianal area (yellow arrowheads) (**A**). Necrotic tissue extended to the scrotum (**B**) and left inguinal region (**C**).

**Fig. 2:**
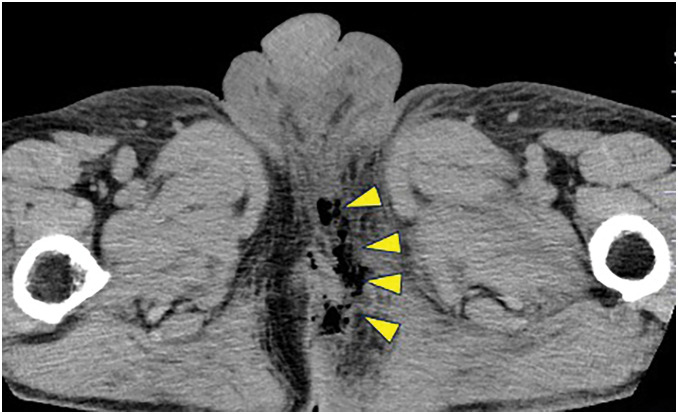
Initial plain CT findings. Edematous changes with gas formation in the soft tissues were observed on the left side of the anus (yellow arrowheads).

Physical examination revealed marked erythema and swelling extending from the perianal area (0–6 o’clock direction) toward the left inguinal region. Blackened skin with central necrosis and skin defects was observed at the 3 and 6 o’clock positions around the anus (**Fig. 1A**). Additionally, a patchy black discoloration was observed on the scrotal skin (**Fig. 1B**), with necrotic tissue extending from the left inguinal region to the left thigh (**Fig. 1C**). Laboratory tests revealed marked inflammation, with a white blood cell count of 16200/mm^3^, C-reactive protein level of 26.1 mg/dL, and procalcitonin level of 11.3 ng/mL. CT revealed edematous changes and the presence of air in the soft tissue on the left side of the anus (**Fig. 2**). Accordingly, FG originating from a perianal abscess extending to the perineum, left thigh, and left inguinal regions was diagnosed. On admission, the blood glucose level was 181 mg/dL and HbA1c was 5.8%, indicating no evidence of diabetes. The FGSI and UFGSI scores were 8 and 11, respectively.

An emergency surgery was performed on the same day. Necrotic skin at the 3 and 6 o’clock positions around the anus was excised, and a thorough palpation was conducted to identify the primary source of infection. The wounds at the 3 and 6 o’clock positions were found to communicate beneath the external anal sphincter. Furthermore, a finger could be easily inserted from the 3 o’clock wound into the deep subcutaneous tissues of the thigh and perineum. These findings indicated a horseshoe-shaped abscess extending posteriorly and to the left of the anus as the primary focus of infection, which had spread and led to FG.

**Fig. 3:**
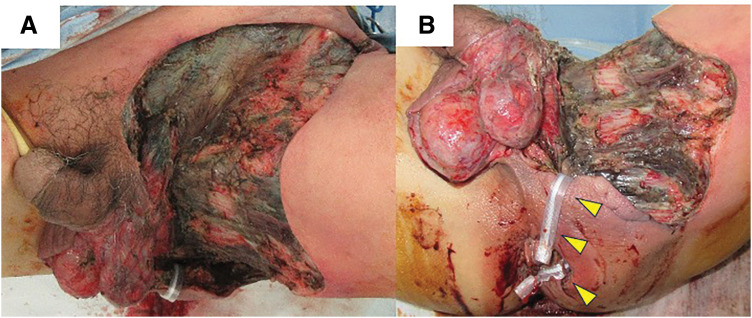
Necrotic tissue in the left inguinal region, scrotum, and perineum was extensively excised (**A**), and a loop drainage technique using Penrose drains was applied after removing the necrotic tissue at the 3 and 6 o’clock positions around the anus (yellow arrowheads) (**B**).

Necrotic tissue was extensively debrided, including the skin of the left scrotum, left inguinal region, and left thigh. Extensive debridement around the anus was avoided to preserve the anal sphincter function. Instead, a loop drainage technique using Penrose drains was employed between the 6 and 3 o’clock positions to manage the horseshoe abscess, following the principles of the seton method. A similar loop drain was also used between the 3 o’clock wound and the debrided area (**Fig. 3A**, **3B**). Notably, meropenem hydrate antibiotic therapy was initiated upon admission. Although blood cultures were negative, wound cultures yielded *Staphylococcus warneri* and *Peptostreptococcus anaerobius*. Based on susceptibility testing, the antibiotic regimen was switched to cefazolin.

**Fig. 4:**
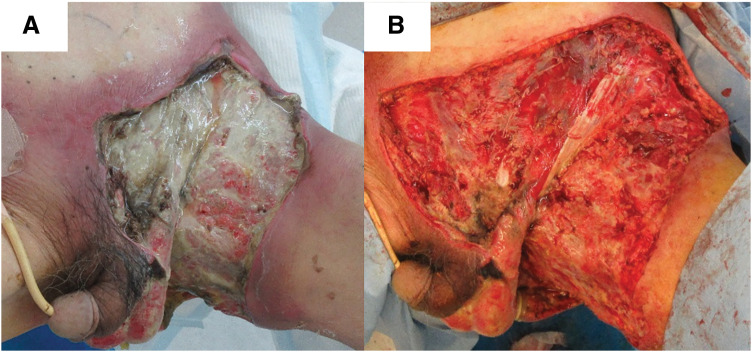
Redness and necrosis of the skin around the wound increased before second debridement on day 10 (**A**). Additional debridement was performed on the thigh and lower abdominal wounds (**B**).

Following the initial surgery, the wound was lavaged daily with warm water and applied sulfadiazine silver cream. On POD 10, despite the laboratory data improvement, with a white blood cell count of 8200/mm^3^ and C-reactive protein level of 7.28 mg/dL, redness and necrosis of the skin around the wound increased. Additional debridement was performed on the thigh and lower abdominal wounds; however, no further debridement was necessary for the perianal and perineal regions, where infection was successfully controlled without additional intervention (**Fig. 4A**, **4B**). Pain was well managed using fentanyl citrate tape for 1 day 0.5 mg for 20 days, and perineal cleaning with warm water was feasible with the drains in place, minimizing wound contamination.

**Fig. 5:**
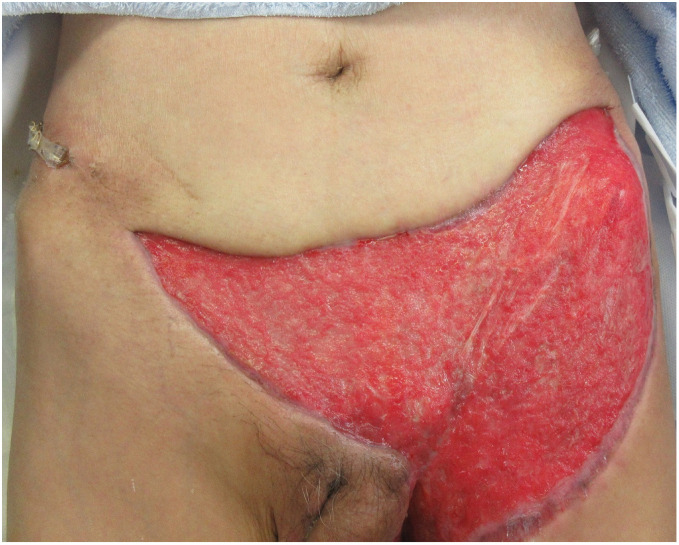
Infection was controlled before skin graft on POD 72.

Once infection was controlled (**Fig. 5**), a full-thickness skin graft was performed on POD 72, and the two Penrose drains, which were never replaced, were removed on day 81. The patient was discharged on day 107, and all the wound was epithelialized on day 148. At discharge, both Low Anterior Resection Syndrome (LARS) and Wexner scores were 0, indicating no impairment of anal or defecatory function. The patient maintained a good QOL for approximately 3 years without defecation disorder or recurrence of infection.

## DISCUSSION

FG was first reported in 1883 as an idiopathic gangrene of the perineal region in previously healthy young men, characterized by acute onset and rapid progression. Recently, FG is recognized as a rare and fatal form of necrotizing fasciitis of the perianal, perineal, and genital regions. Risk factors include diabetes, human immunodeficiency virus infection, alcoholism, and other immune-compromised states. FG wound cultures typically yield multiple microorganisms, including anaerobic bacteria and *Streptococcus, Staphylococcus*, and *Escherichia* being the commonly identified species. Despite aggressive treatment, the current mortality rate for FG is approximately 20%–40%.^1–5)^ Reported etiologies include genitourinary (24%), anorectal (24%), intra-abdominal (10%), traumatic (2%), and undetermined (38%) causes.^5)^

Following the early recognition of FG, broad-spectrum antibiotics, resuscitation, and aggressive debridement are the standard treatment.^2–5)^ The primary principle of FG treatment is urgent initial surgical debridement, involving the complete removal of necrotic tissue until viable tissue is identified.^2,3)^ To prevent death, immediate incision, drainage, and complete debridement, regardless of form and function, are recommended.^5)^ Following the initial debridement, close wound monitoring and repeated debridement are necessary to control infection.^2,3)^ Although complete surgical debridement is generally recommended, it frequently affects large areas, resulting in significant deficits.^3)^

**Fig. 6:**
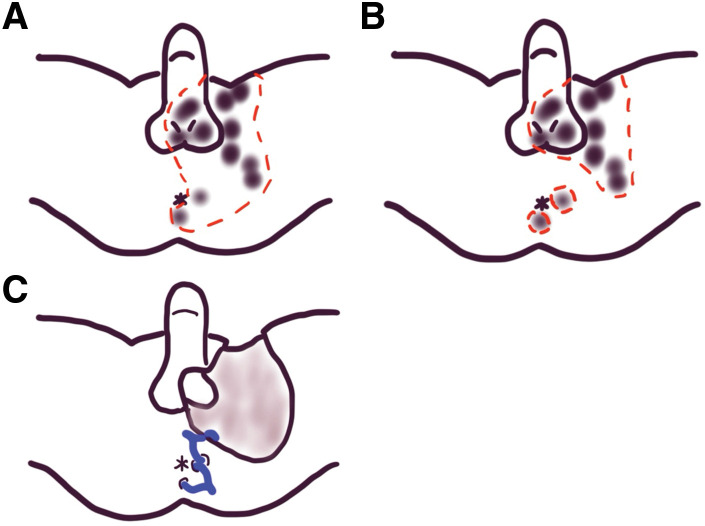
Proposed resection line for extensive debridement (**A**). To preserve anal function, debridement was performed to a limited area (**B**), and two loop drains were placed around the anus (**C**).

In the present case, the left unilateral horseshoe abscess was considered the primary infectious focus, and the necrotizing infection spread from the abscess to the perineal, genital, thigh, and inguinal regions. Complete debridement of this gangrene required en bloc resection of the skin in the perianal, perineal, genital, thigh, and lower abdominal regions (**Fig. 6A**). To prevent anal dysfunction, loop drainage using Penrose drains was performed on the horseshoe abscess instead of complete debridement of the perianal region (**Fig. 6B**). A Penrose drain was placed between the posterior midline wound and left lateral wound through the deep postanal space, similar to a drainage seton—a drainage method for managing horseshoe abscesses.^6)^ Meanwhile, aggressive debridement was performed on the genital, thigh, and left inguinal regions, as debridement in these regions may result in few functional disturbances. Another Penrose drain was placed between the left lateral wound and the debridement wound to drain the perineal region (**Fig. 6C**).

The loop drainage technique has been proposed as an alternative, less-invasive approach for abscess management.^7)^ As our patient reported minimal perianal pain postoperatively, perianal wound care and postoperative physical rehabilitation could be easily performed. Patients treated with the loop drainage technique experience significantly less pain and higher satisfaction.^8)^ However, due to lower invasiveness, the loop drainage technique may not always provide sufficient infection control. Therefore, close wound monitoring remains essential, and additional debridement should be performed immediately, if necessary, after loop drainage.

In 1995, Laor et al.^9)^ introduced the FGSI as a tool for assessing FG severity, reporting 75% and 22% mortality rates for the scores of ≥10 and ≤9, respectively. Yilmazlar et al.^10)^ reported a 94% probability of death with a UFGSI score of >9 and an 81% probability of survival with a UFGSI score of ≤9. The FGSI is calculated based on parameters such as vital signs and laboratory test results. The UFGSI expands upon the FGSI by incorporating the extent of necrotic tissue involvement and patient age. The total UFGSI score ranges from 0 to 36, with higher scores indicating more severe disease and a higher risk of mortality. In the present case, the FGSI and UFGSI scores were 8 and 11, respectively. Based on the scores, this case was diagnosed as FG with extensive local spread.

Chen et al.^11)^ reported a minimal and intermittent cutting debridement technique with a draining seton between every two incisions rather than extensive debridement for the treatment of FG. The average FGSI score was 1.86, and they concluded that their technique resulted in lower mortality and reduced soft tissue destruction in the treatment of patients with limited FG. Because this case involved widespread FG, extensive and aggressive debridement was required. Therefore, loop drainage using Penrose drains, which may be similar to the technique reported by Chen, et al., was performed only around the anus. By performing the loop drainage instead of aggressive debridement, perianal skin, superficial external anal sphincter muscle and left pudendal nerve might be preserved, and anal function was maintained.

The Wexner score, now commonly referred to as the Cleveland Clinic Florida Fecal Incontinence Score (CCFIS), is used to evaluate the severity of fecal incontinence. The total score ranges from 0 to 20, with higher scores indicating more severe incontinence.^12)^ The LARS score is used to assess bowel dysfunction following low anterior resection. It ranges from 0 to 42, with higher scores indicating more severe symptoms, and is categorized as no LARS (0–20), minor LARS (21–29), and major LARS (30–42).^13)^ Using our surgical approach, both the LARS and CCFIS scores, as well as patient-reported symptoms, demonstrated preserved anal function during long-term postoperative follow-up.

Although this study has a limitation of a single case report, the combination of aggressive debridement and loop drainage technique may be effective, and the loop drainage technique could be performed in a limited area for extensive FG cases.

## CONCLUSIONS

This study reports a case of FG treated with a loop drainage technique around the anus and aggressive surgical debridement of the genital, thigh, and inguinal regions while preserving the postoperative anal function. The loop drainage technique in the perianal region may preserve the postoperative anal function. Notably, the loop drainage technique can be considered a therapeutic option in areas where aggressive debridement may cause functional impairment.

## DECLARATIONS

### Availability of data and materials

The datasets supporting the conclusions of this article are included within the article.
